# Liver Mucinous Cystic Neoplasm With Obstructive Jaundice

**DOI:** 10.7759/cureus.31970

**Published:** 2022-11-28

**Authors:** Mohammad N Alzoubi, Rahaf B Alhendi, Ayah A Eyalawwad, Khaled I Daradka, Badi A Rawashdeh

**Affiliations:** 1 Department of General Surgery, The University of Jordan, Amman, JOR; 2 Transplant Surgery, Medical College of Wisconsin Green Bay, Milwaukee, USA

**Keywords:** hepatectomy, laparoscopic technique, obstructive jaundice, mucinous cystic neoplasm, liver cyst

## Abstract

Biliary mucinous cystic neoplasms (BMCNs) are rare and slow-growing lesions that are usually discovered incidentally. They can imitate various other liver tumors. Here, we present a 31-year-old female patient who presented with complaints of abdominal pain, nausea, shortness of breath, and obstructive jaundice. Ultrasound showed a large, lobulated, cystic liver mass. Abdominal computed tomography (CT) scan showed features suggestive of a hydatid cyst or complicated liver cyst. A laparoscopic deroofing was performed and showed a liver cyst involving segments 2, 3, 4A, and 4B. Histopathology showed that the cyst wall was lined by columnar mucin-producing epithelium with multifocal areas of ovarian-like stroma, and the diagnosis of biliary mucinous cystic neoplasms was made. A one-year, follow-up radiological examination did not show any recurrence.

BMCNs are quite rare. The nonspecific nature of the symptoms and radiological characteristics makes the diagnosis of BMCN challenging. Imaging modalities can aid in the diagnosis, but pathological examination is essential in confirming a definite diagnosis.

## Introduction

Mucinous cysts are complex, multiseptated cysts in which mural thickening, nodularity, debris-containing fluid, and hemorrhagic or protein-containing materials are frequently observed [1]. Biliary mucinous cystic neoplasm (BMCN) is one of those mucinous cysts and is a rare benign cystic neoplasm of the biliary system, with an estimated incidence of about 5% of all hepatobiliary cystic neoplasms [2]. BMCNs can present with a unique site other than their usual presentation and can communicate with the bile duct [3]. They occur almost exclusively (85-95%) in middle-aged females [4]. The prevalence of hepatic cysts is increasing as a result of the increased availability and use of abdominal imaging modalities. However, a precise preop diagnosis of BMCNs can be daunting since it resembles multiple other lesions of the liver, especially complex cysts [1]. The presence of septations, the relationship of the septation to the cyst wall, and septal enhancement were sensitive imaging features in the detection of BMCN [5]. The defining feature is the development of a mesenchymal ovarian-type stroma consisting of a thick layer of compact, spindle-shaped cells beneath the lining epithelium [6].

## Case presentation

A 31-year-old Arabic female presented with jaundice, right upper quadrant pain, and nausea that was associated with loss of appetite, shortness of breath, and pleuritic chest pain. The abdominal pain was in the right upper quadrant, colicky in nature, and radiating to the back. The patient noticed yellowish discoloration of the eyes and face. On examination, the abdomen was soft and distended. Massive tender hepatomegaly was noted reaching the right lumbar and epigastric regions. The patient is a nonsmoker and doesn’t drink alcohol or use any substances. Laboratory tests revealed abnormal liver function tests (Table 1).

**Table 1:** Summary of the laboratory values on presentation ALT - alanine transaminase; AST - aspartate transaminase; GGT - gamma-glutamyl transferase; LDH - lactate dehydrogenase; CEA - carcinoembryonic antigen; AFP - alpha-fetoprotein

Labs	Unit	Result	Reference Range
Total protein	g/dl	7.3	6.0 -8.3
Albumin	gm/dl	4.1	3.4-5
Total bilirubin	µmol/L	70.13	1.71 to 20.5
Direct bilirubin	µmol/L	59.86	<5.1
Indirect bilirubin	µmol/L	10.26	3.4-12.0
ALT	U/L	197	1-65
AST	U/L	179	1-37
GGT	U/L	218	15-85
LDH	U/L	384	140-280
Alkaline phosphatase	U/L	351	39-119
CEA	ng/mL	LESS THAN 0.00	<5.0
AFP	ng/mL	3.8	<20.0
Serology for ECCHINO		Negative	Negative

**Figure 1:**
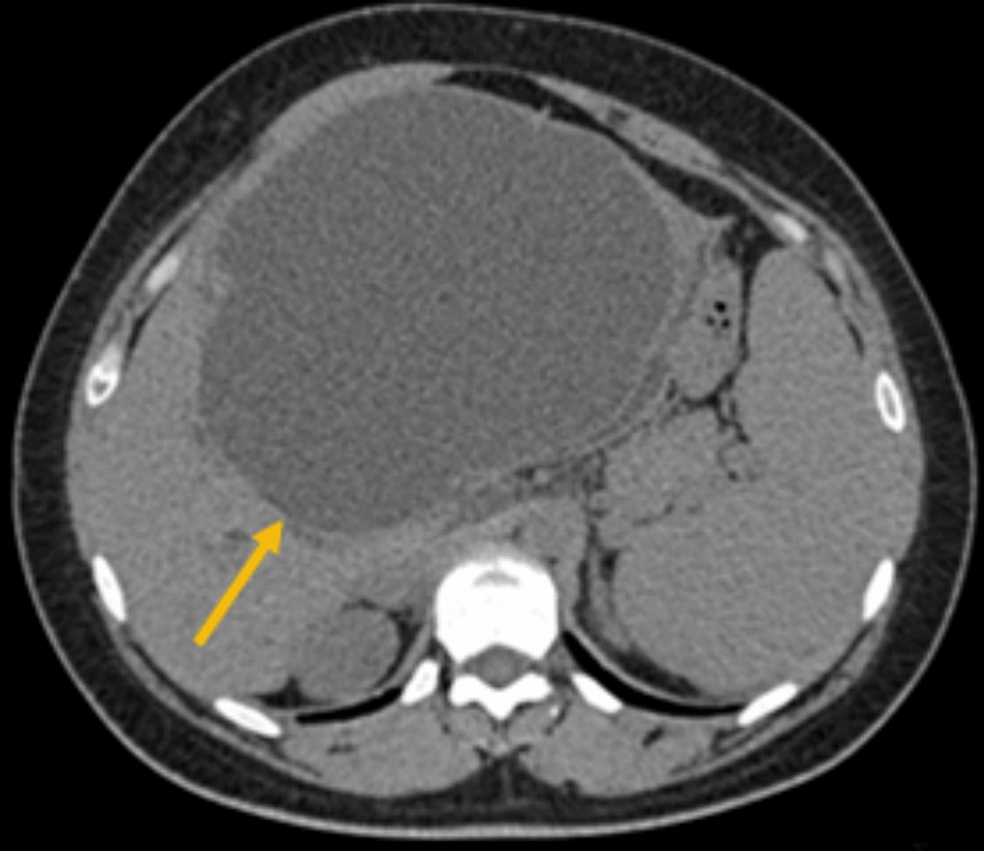
Axial view of computed tomography (CT) of the abdomen The CT scan shows a large hepatic cyst measured 13*17*20 cm seen arising from the left hepatic lobe with a double-wall sign and suspicion of focus of calcification.

Ultrasonography exam showed a large, lobulated cystic mass with low-level internal echoes originating from the liver at the porta hepatis measuring 13*17.5*18.5 cm associated with mild dilatation of the intrahepatic biliary tree. An abdominal computed tomography (CT) scan showed a large hepatic cyst measured 13*17*20 cm seen arising from the left hepatic lobe with a double-wall sign and suspicion of focus of calcification. However, no abnormal enhancement was seen. The spleen measured 15 cm, without a focal lesion, and the right ovarian cyst with blood or fluid level noted measured 2.7 cm (Figure 1).

**Figure 2:**
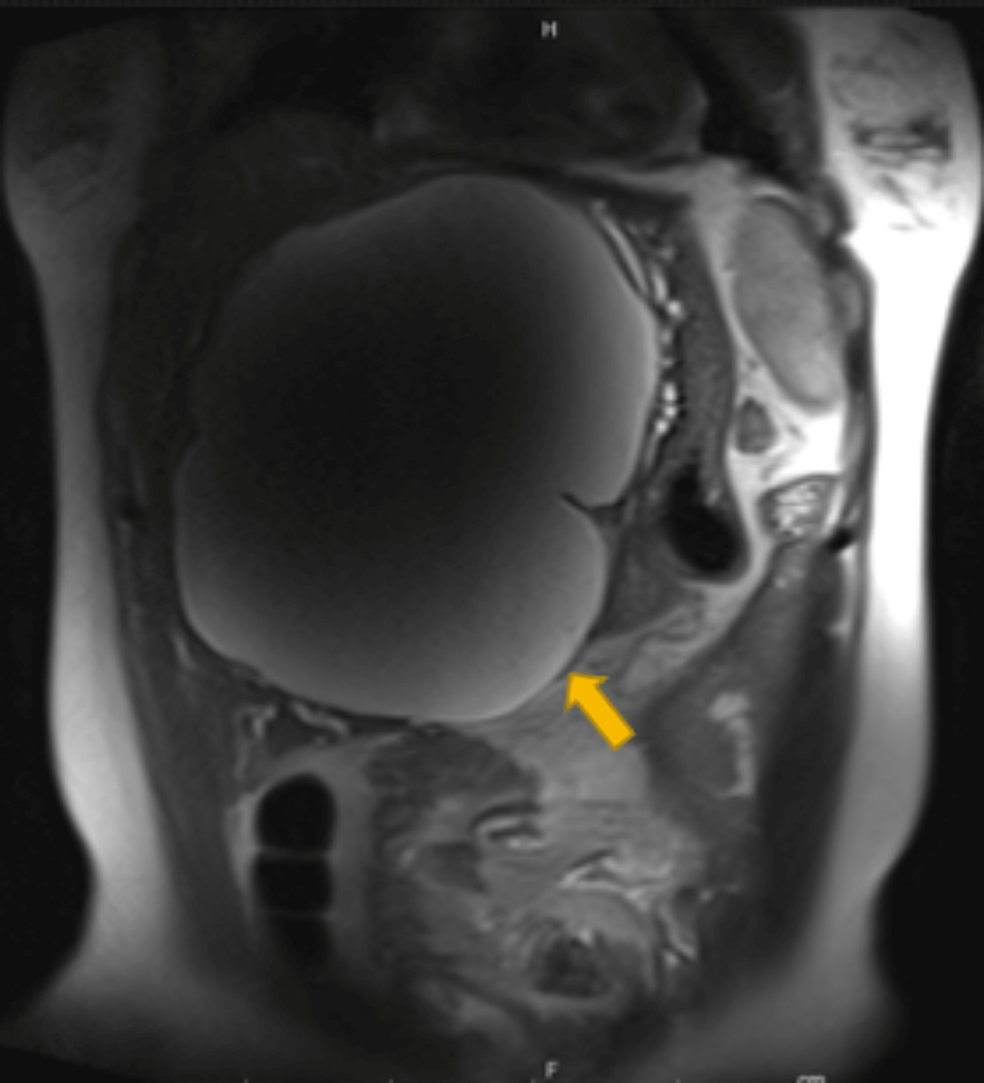
sagittal view of Magnetic resonance cholangiopancreatography (MRCP) without contrast MRCP showed a large right upper quadrant cyst without internal soft tissue nodules or membranous part

Magnetic resonance cholangiopancreatography (MRCP) without contrast showed a large, right, upper quadrant cyst without internal soft tissue nodules or membranous part causing compression on the adjacent structures in addition to the intrahepatic biliary tree (Figure 2).

The patient underwent laparoscopic surgery, during which the cyst roofing was partially removed. An examination of the cyst cavity did not reveal any evidence of a hydatid cyst, and a specimen was sent for histological examination.

**Figure 3:**
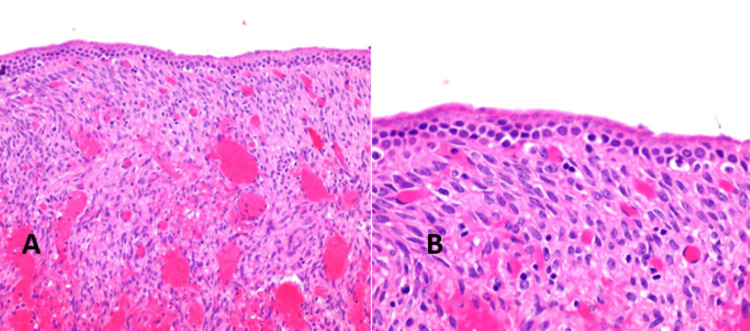
A: The hematoxylin-eosin (x 20)-stained histologic section. Reactive changes in the form of hemorrhage, edema, congestion, and fibrosis were noted. B: High magnification revealed the pathognomic columnar mucinous epithelial cells with underlying ovarian-type stroma. Hematoxylin & Eosin, 40X

A microscopy exam showed a cyst wall lined by columnar, mucin-producing epithelium, and the underlying stroma shows multifocal areas of ovarian-like stroma with ovoid to spindle cells that are immunoreactive to the estrogen receptor, progesterone receptor, and inhibin (focal weak) immunohistochemical stains. The histopathological features were consistent with mucinous cystic neoplasm (Figure 3).

However, these lesions are described to be multilocular; the examined sections show no evidence of high-grade dysplasia or invasive malignancy. The liver's non-invasive mucinous cyst tumor was confirmed histologically. Following the procedure, there were no complications, and a year later, the patient showed no signs of recurrence.

## Discussion

Cystic liver lesions are a diverse collection of diseases with varying origins, prevalence, and clinical symptoms. The majority of liver cysts that are incidentally found are benign [7]. Malignant transformation in the case of a mucinous cystic neoplasm (cystadenoma) or anaphylactic shock in the case of a hydatid cyst are examples of problems that can occur with specific types of hepatic cysts [7]. Some of these problems may require surgical intervention on rare occasions. Upper abdominal heaviness, stomach discomfort or pain, and anorexia were the most frequent symptoms. These symptoms occurred in some individuals several years before they were diagnosed. Numerous individuals were nonetheless asymptomatic, with lesions accidentally discovered in tests using abdominal imaging. Rarely, when there is a biliary obstruction, individuals suffer from colicky discomfort or jaundice, like in our case, due to tumor extension to the biliary confluence. The differential diagnoses include intraductal papillary neoplasm of the bile duct (IPNB), mucinous cystic neoplasm (MCN), simple hepatic cysts, pyogenesis of the liver, hydatid cysts, and degeneration of the liver by any tumor [8]. Laboratory investigations can aid in distinguishing between biliary cystadenoma from an infected cyst based on leukocytosis and positive amebic and echinococcal serology. High cholestatic values are related to biliary tree obstruction or compression. Some authors proposed higher standards of CA 19-9 (serum and cyst fluid) to differentiate BMCN from other cystic lesions [9]. For a definite diagnosis, a histological evaluation is necessary, but an imaging study might suspect the lesion. The differential diagnosis includes MCN with concomitant invasive carcinoma (cystadenocarcinoma). The absence of septations and papillary projections and the presence of serous cystic fluid is typically distinguishing between simple and complex cysts.

Calcifications, in addition to positive serology tests, are often linked with echinococcal cysts [10]. Microscopically, mucinous cystic neoplasms (MCNs) are bordered with a cuboidal or columnar epithelial, biliary-type, mucus-secreting, supporting, thick, fibrous cellular stroma similar to the ovarian tissue MCNs. A loose and less cellular collagen layer surrounds the lining [11]. Magnetic resonance imaging (MRI) provides the extra benefit of revealing anatomic connections inside the liver to a greater extent than CT, which can help in surgical planning. MRI findings vary depending on the kind of weighted imaging used, with T1 scans displaying variable cystic fluid signal intensities and T2-weighted images revealing intracystic septations. MRI may be useful in delineating biliary obstructions and biliary communications with the cyst. Endoscopic retrograde cholangiopancreatography may be used in rare cases to sample tissue from lesions interacting with the biliary network. As pre-malignant lesions are deemed mucinous cystadenomas, complete surgical ablation is the proper therapy with fewer than 10% recurrence [12].

To conclude, biliary cystadenomas are rare tumors and should be examined with multilocular, hepatic cystic lesions, particularly in patients with obstructive jaundice, especially in women. The therapy of choice is complete excision due to its potential malignant transformation and its natural history of gradually increasing and recurring after partial excision.

## Conclusions

We present a case of a young lady in which BMCN caused the narrowing of the bile duct with the presentation of abdominal pain and jaundice. BMCN with extrahepatic biliary tract involvement is a rare, slow-growing tumor that arises more commonly in women and has the potential for malignant transformation. Clinically, it presents with a prolonged course of epigastric fullness later followed by obstructive jaundice.

In summary, BMCN may present with abdominal pain and features of obstructive jaundice. Clinical presentation, complemented with imaging and histopathological evaluation, is essential for diagnosis. Complete surgical resection is the treatment of choice to prevent recurrence and malignant transformation.
